# Case Report: Early Detection of Carotid Artery Stenosis with Falck Multifunctional Device (FMD), A Revised Ophthalmodynamometry Method

**DOI:** 10.12688/f1000research.164433.1

**Published:** 2025-05-21

**Authors:** Brian Murillo, Anny M.S. Cheng, Sandeep Samudre, John T LiVecchi, Shailesh K. Gupta

**Affiliations:** 1 New York Medical College School of Medicine, Valhalla, New York, USA; 2 Ophthalmology, Broward Health North, Deerfield Beach, Florida, 33064, USA; 3 Ophthalmology, University of Florida, Gainesville, FL, 32605, USA; 4 Ophthalmology, Advanced Research Center, Deerfield Beach, FL, 33064, USA

**Keywords:** carotid artery stenosis, internal carotid artery, mean central retinal artery pressure, ocular perfusion pressure, Ophthalmodynamometry

## Abstract

Advanced clinical diagnostic tools enable ophthalmologists to diagnose not only ocular pathologies but also identify disorders that extend beyond ocular diseases. Ophthalmodynamometry (ODM), a screening tool that most ophthalmologists do not commonly use, measured reduced mean central retinal artery pressure (MCRAP) in the clinical setting. We describe a 70-year-old female with a reduced MCRAP in the right eye who identified 50% stenosis in her right internal carotid artery (ICA). Early diagnosis facilitated prompt management and potentially prevented future ischemic events.

## Introduction

Ophthalmodynamometry (ODM) is a non-invasive method to evaluate ophthalmic vascular pressure dynamics in the central retinal artery (CRA). This technique involves increasing the intraocular pressure (IOP) by applying a standardized pressure to the globe. CRA pressure is measured at the point where the lowest standardized pressure induces pulsations. ^1, 2^ ODM assessment of CRA perfusion provides insight into other arteries due to their direct anatomical communication. When ODM measures mean central retinal artery pressure (MCRAP), the ocular perfusion pressure (OPP) can be derived by calculating the difference between MCRAP and IOP. ^3^ Because of the anatomical relationship between the CRA, the ophthalmic artery (OA) and internal carotid artery (ICA), narrowing in the OA or the ICA will decrease MCRAP and/or OPP. Therefore, identifying compromised MCRAP or reduced OPP through ODM may suggest systemic cerebrovascular occlusive disease, ^4^ prompting further vascular assessments.

ODM has traditionally been measured using various techniques of raising the IOP, including compression or negative suction pressure. Many of these methods often require a second operator to visualize central artery changes. Recently, the Falck Multifunctional Device (FMD, Falck Medical, CT, USA) has modernized the classic ODM approach by incorporating a digitalized pressure sensor into the holding grip of a slit lamp. This improvement allows a calibrated pressure application to induce fine vascular pulsations in the CRA, eliminating the need for visualizing vessel changes during traditional ODM techniques. In this report, we present one of the first cases of FMD utility in an asymptomatic individual with minimal vascular risk factors. The patient presented with diminished MCRAP and was identified as having focal carotid vascular occlusive disease on further carotid doppler testing. Written informed consent was obtained from the patient to publish this report.

## Case history

A 70-year-old female in good general health, except with a history of well-controlled hypertension presented for a routine eye exam. She reported no negative effect of taking amlodipine and losartan. Her family history was significant for coronary artery disease, but she was non-diabetic and non-smoking. Her past medical history and surgical history was otherwise unremarkable. Her best corrected visual acuity was 20/20 OU. Slit lamp examination of the anterior segment was unremarkable. Bilateral dilated indirect fundoscopic examination showed arterio-venous nicking, but the retinal nerve fiber layer could be seen clearly. The optic disc margin was sharp and there was no retinal hemorrhage or exudate.

## Methods

ODM using the FMD was performed because of the presence of vascular risk factors. FMD ODM directly measured the following: IOP of 15 mmHg OD and 14 mmHg OS; and MCRAP of 51.6 mmHg OD and 55.4 mmHg OS. Brachial mean arterial pressure (MAP) measured with automated blood pressure cuff was 102.7 mmHg on right and 93.3 mmHg on left. The right MCRAP (51.6 mmHg), representing 50% of the right MAP, was significantly lower (variability 3%) than the left MCRAP (55.4 mmHg), representing 59% of the left MAP. Similarly, the OPP revealed significantly lower pressure (variability 2%) in the right eye of 36.7 mmHg than in the left eye of 41.2 mmHg. The reduced MCRAP and OPP OD prompted further evaluation with carotid artery duplex scan.

### Outcome and follow-up

**Figure 1.**
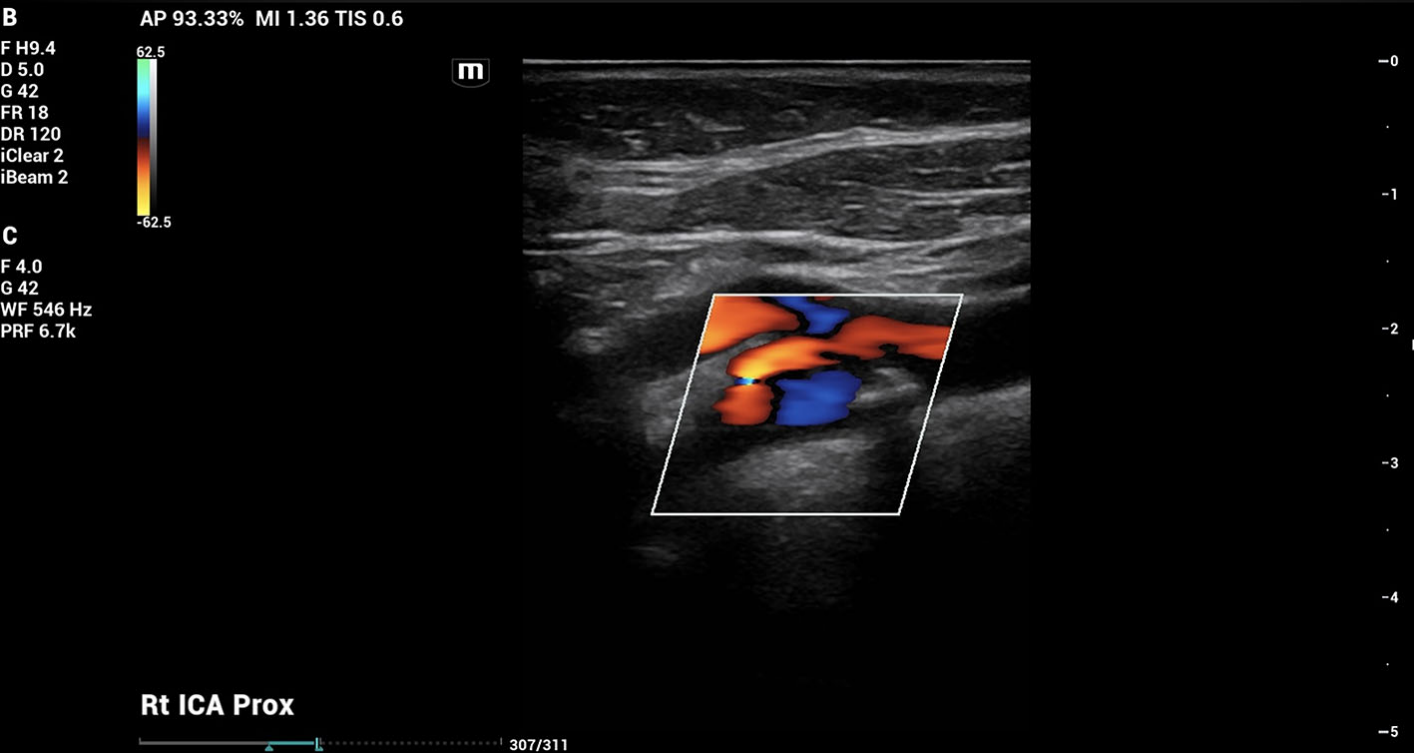
Carotid doppler imaging. Carotid doppler showed 50% stenosis in right carotid bulb and proximal cervical internal carotid artery (ICA).

On doppler imaging study, all vessel velocities measured by carotid duplex were significantly higher on the right side than the left. The measured peak velocity of the right internal carotid artery (ICA) was 122.02 cm/s, the external carotid artery (ECA) 118.45 cm/s, and the common carotid artery (CCA) 98.81 cm/s. The left sided measured peak velocities were the ICA of 47.33 cm/s, the ECA of 75.59 cm/s, and the CCA of 68.88 cm/s. In addition, the vessel velocity difference was greater in the ICA at 74.69 cm/s, than in the ECA (42.86 cm/s) or the CCA (29.93 cm/s). The carotid duplex interpretation concluded 50% stenosis in the right ICA ( Figure 1) with heterogenous calcified plaquing and minimal stenosis on the left. The patient was therefore referred for further vascular evaluation.

## Discussion

In this report, ODM using the FMD demonstrated reduced MCRAP and OPP in an otherwise asymptomatic patient. The patient had carotid artery stenosis as determined by carotid duplex ultrasound. Both the right MCRAP and OPP were not only measured low, but also considerably lower than the left MCRAP and OPP readings, suggesting that these parameters can be significantly and linearly linked with the existence and severity of carotid artery stenosis. ^5^

Compared to classic ODM, FMD averages multiple measurements over the cardiac cycle, making it more reproducible. The repeated measurement for each eye in our patient was consistent, with a low variation of 2 to 3%. The FMD can also be performed by a single operator as it is mounted to a slit-lamp thereby reducing variability. FMD has the potential to be used in a variety of applications. For example, it can detect patients at risk of having a drop in OPP after anti-VEGF injections, improving safety. ^6^ The FMD can also measure accurate and repeatable applanation forces that compensate for the effect of corneal thickness and curvature. ^7^ FMD can be a promising diagnostic tool for glaucoma screening and glaucoma severity assessment. ^8^ Our case showed that the FMD may also be superior to relying on historical vascular risk factors alone in determining the need for further vascular testing.

Collectively, these findings render ODM using the FMD as a relatively fast, inexpensive, and reproducible office-based diagnostic test that can help determine a patient’s risk profile for systemic cerebrovascular disease. The association between reduced MCRAP and OPP and carotid artery stenosis suggests that FMD assisted ODM should be further explored as an indicator of compromised cerebrovascular hemodynamic status. Thus, the FMD provides ophthalmologists with an inexpensive non-invasive screening tool for cardiovascular disease.

## Institutional review board statement

Institutional approval was waived as our single case report involves retrospective medical record review of one patient and the only interaction with the patient has been for purposes of treating the patient and does not meet the Common Rule definition of research (45 CFR 164.501).

## Informed consent statement

Written informed consent for publication of her clinical details and/or clinical images was obtained from the patient.

## Reporting guidelines

Mendeley Data: Murillo, Brian; Cheng, Anny; Samudre, Sandeep; LiVecchi, John; Gupta, Shailesh (2025), “Early Detection of Carotid Artery Stenosis with Falck Multifunctional Device (FMD), A Revised Ophthalmodynamometry Method”, DOI: 10.17632/893knw22df.1 ^9^

The project contains the following reporting guidelines:

1. CARE_checklist.pdf

Data are available under the terms of the Creative Commons Attribution 4.0 International license (CC-BY 4.0).

## Data Availability

All data underlying the results are available as part of the article and no additional source data are required.

## References

[1] Best M Blumenthal M Futterman HA : Critical closure of intraocular blood vessels. *Arch. Ophthalmol.* 1969;82(3):385–392. 10.1001/archopht.1969.00990020387018 5806063

[2] Krieglstein GK Silva FA da : Comparative measurements of the ophthalmic arterial pressure using the mikuni dynamometer and the stepanik-arteriotonograph®. *Albrecht Von Graefes Arch. Klin. Exp. Ophthalmol.* 1979;212:77–91. 10.1007/BF00587599 317630

[3] Jonas JB : Reproducibility of ophthalmodynamometric measurements of central retinal artery and vein collapse pressure. *Br. J. Ophthalmol.* 2003;87:577–579. 10.1136/bjo.87.5.577 12714398 PMC1771675

[4] Paulson OB : Ophthalmodynamometry in internal carotid artery occlusion. *Stroke.* 1976;7(6):564–566. 10.1161/01.STR.7.6.564 1006729

[5] Murillo BA Cheng AM Tsai J : Decreased Ocular Perfusion Pressure Associated With Reverse Ophthalmic Artery Flow on Transcranial Doppler Ultrasonography. *Cureus.* 2024;16(5):e60706. 10.7759/cureus.60706 38899251 PMC11186674

[6] Reichel E Shah H : The Effects of Intravitreal Anti-VEGF Injections on Ocular Perfusion Pressure. *Invest. Ophthalmol. Vis. Sci.* 2010;51:5032.

[7] Falck FY Falck R : *Clinical and Manometric Comparison of The Falck Autotonometer and The Goldmann Tonometer. Annual Meeting of the Association for Research in Vision and Ophthalmology (ARVO).* Fort Lauderdale, Florida:2008 Apr 27.

[8] Falck FY Falck R : *Clinical Comparison of FAT1 to Calibrated Weight Indentation Tonography. Annual Meeting of the Association for Research in Vision and Ophthalmology (ARVO).* Fort Lauderdale, Florida:2011 Jan 10.

[9] Murillo B Cheng A Samudre S : Early Detection of Carotid Artery Stenosis with Falck Multifunctional Device (FMD), A Revised Ophthalmodynamometry Method. *Mendeley Data.* 2025;V1. 10.17632/893knw22df.1 PMC12529650 41112926

