# Supplementary material for: Case Report: Early Detection of Carotid Artery Stenosis with Falck Multifunctional Device (FMD), A Revised Ophthalmodynamometry Method
Source: F1000Res. 2025 Oct 9;14:508. Originally published 2025 May 21. [Version 2] doi: 10.12688/f1000research.164433.2 (PMC12529650; doi:10.12688/f1000research.164433.2)
Supplement: Peer review history

# Reviewer report: Reviewer response for version 2

**DOI:** 10.5256/f1000research.189354.r421992

**Published:** 2025-11-06

**Authors:** Andrew Tirsi

**Affiliations:** 1 Donald and Barbara Zucker School of Medicine at Hofstra/Northwell, Hempstead, New York, USA

No further comments to make

Are enough details provided of any physical examination and diagnostic tests, treatment given and outcomes?

Yes

Is the case presented with sufficient detail to be useful for other practitioners?

Yes

Is sufficient discussion included of the importance of the findings and their relevance to future understanding of disease processes, diagnosis or treatment?

Yes

Is the background of the case’s history and progression described in sufficient detail?

Yes

Reviewer Expertise:

Normal tension glaucoma, OPP, electrophysiology in glaucoma, retinal ganglion cell dysfunction in glaucoma, mitochondrial dysfunction in glaucoma and other retinal diseases

I confirm that I have read this submission and believe that I have an appropriate level of expertise to confirm that it is of an acceptable scientific standard.

# Reviewer report: Reviewer response for version 2

**DOI:** 10.5256/f1000research.189354.r421993

**Published:** 2025-10-14

**Authors:** Efe Kanter

**Affiliations:** 1 Izmir Katip Çelebi University, Izmir, Turkey

Article is ready for indexing.

Are enough details provided of any physical examination and diagnostic tests, treatment given and outcomes?

Yes

Is the case presented with sufficient detail to be useful for other practitioners?

Yes

Is sufficient discussion included of the importance of the findings and their relevance to future understanding of disease processes, diagnosis or treatment?

Yes

Is the background of the case’s history and progression described in sufficient detail?

Yes

Reviewer Expertise:

Emergency medicine, ultrasonography, novel methodology in stroke, non-invasive ophthalmic monitoring.

I confirm that I have read this submission and believe that I have an appropriate level of expertise to confirm that it is of an acceptable scientific standard.

# Reviewer report: Reviewer response for version 1

**DOI:** 10.5256/f1000research.180947.r414795

**Published:** 2025-09-30

**Authors:** Efe Kanter

**Affiliations:** 1 Izmir Katip Çelebi University, Izmir, Turkey

This case report presents the early detection of internal carotid artery stenosis in an asymptomatic patient through ophthalmodynamometry (ODM) using the Falck Multifunctional Device (FMD). The manuscript is original and clinically relevant, as it demonstrates the potential of a relatively underutilized ophthalmic tool in identifying systemic cerebrovascular risk. The description of the case is sufficiently detailed, with well-documented examination findings and comparative measurements that convincingly support the diagnosis.

 The report could be further strengthened by addressing a few minor issues. The possible impact of antihypertensive therapy, particularly amlodipine, on ocular perfusion pressure should be briefly discussed, as well as whether the patient had risk factors such as obstructive sleep apnea that might influence ODM measurements. Including a figure or schematic showing the device display and measurement process would add clarity for readers unfamiliar with the FMD. Furthermore, the discussion would benefit from linking the findings to recent literature on ocular hemodynamic parameters as predictors of cerebrovascular events. In this context, the study by Kanter E. et al. [Reference 1] provides supportive evidence for the prognostic value of ophthalmic artery Doppler ultrasound in ischemic and hemorrhagic stroke and would enrich the discussion.

Are enough details provided of any physical examination and diagnostic tests, treatment given and outcomes?

Yes

Is the case presented with sufficient detail to be useful for other practitioners?

Yes

Is sufficient discussion included of the importance of the findings and their relevance to future understanding of disease processes, diagnosis or treatment?

Yes

Is the background of the case’s history and progression described in sufficient detail?

Yes

Reviewer Expertise:

Emergency medicine, ultrasonography, novel methodology in stroke, non-invasive ophthalmic monitoring.

I confirm that I have read this submission and believe that I have an appropriate level of expertise to confirm that it is of an acceptable scientific standard, however I have significant reservations, as outlined above.

## References

[1] : A new diagnostic method in ischemic and hemorrhagic stroke: Doppler ultrasound of ophthalmic artery. *Clinical Neurology and Neurosurgery* .2025;254: 10.1016/j.clineuro.2025.108959 10.1016/j.clineuro.2025.108959 40359610

# Response

**Published:** 2025-10-06

**Authors:** Anny Cheng

**Affiliations:** Ophthalmology, University of Florida, Gainesville, Florida, USA

We thank you and the reviewers for your valuable comments. We have revised the manuscript accordingly and provided the following point-to-point responses:

**Reviewer  Comments:**

**Comment :**

 The report could be further strengthened by addressing a few minor issues. The possible impact of antihypertensive therapy, particularly amlodipine, on ocular perfusion pressure should be briefly discussed, as well as whether the patient had risk factors such as obstructive sleep apnea that might influence ODM measurements.

**Reply:** We discussed the effect of Obstructive sleep apnea and amlodipine on OPP in Discussion Section, 2 ^nd^ paragraph “Notably, systemic blood pressure may significantly influence OPP.​  A study reported that a nocturnal dip in blood pressure, characterized by a systolic reduction of 10-12% and a diastolic reduction of 14-17%, has been linked to compromised OPP. ^6^ Patients receiving aggressive antihypertensive medication that leads to excessive blood pressure lowering are also at risk of low OPP. If our patient's systemic blood pressure was not appropriately controlled with amlodipine, it could paradoxically lead to an increase in OPP, potentially masking cardiovascular disease diagnoses. The relationship between OPP and ocular blood flow is complex, largely due to the eye's autoregulation mechanisms. However, vascular endothelial dysfunction, such as that seen in obstructive sleep apnea (OSA), can impair these autoregulatory capabilities, leading to unstable OPP and significantly impacting ocular perfusion. ^7^ “

*6.                 Schmidl D, Garhofer G, Schmetterer L. The complex interaction between ocular perfusion pressure and ocular blood flow - relevance for glaucoma. Exp Eye Res. 2011;93(2):141-155. doi:10.1016/j.exer.2010.09.002*

*7.                 Faridi O, Park SC, Liebmann JM, Ritch R. Glaucoma and obstructive sleep apnoea syndrome. Clin Exp Ophthalmol. 2012;40(4):408-419. doi:10.1111/j.1442-9071.2012.02768.x*

**Comment :**Including a figure or schematic showing the device display and measurement process would add clarity for readers unfamiliar with the FMD.

**Reply:** we added a figure and explanation showing the screen of the device with all the measurements per request.

**Comment : **Furthermore, the discussion would benefit from linking the findings to recent literature on ocular hemodynamic parameters as predictors of cerebrovascular events. In this context, the study by Kanter E. et al. [Reference 1] provides supportive evidence for the prognostic value of ophthalmic artery Doppler ultrasound in ischemic and hemorrhagic stroke and would enrich the discussion.

 1. Kanter E, Payza U, Karakaya Z, Acar H, et al.: A new diagnostic method in ischemic and hemorrhagic stroke: Doppler ultrasound of ophthalmic artery. *Clinical Neurology and Neurosurgery*. 2025; **254**.

**Reply:** We linking our findings to recent literature “Kanter et al 2025” in Discussion Section paragraph 3. “Our findings align with a recent Turkish prospective study that investigated 65 stroke patients (42 ischemic, 23 hemorrhagic) and 27 stroke-free controls, utilizing Doppler ultrasound (OAD-US) to measure peak systolic velocity (PSV) and end-diastolic velocity (EDV) in the ophthalmic artery. The receiver operating characteristic (ROC) analysis revealed high diagnostic accuracy in PSV ratio of 0.913 and the EDV ratio of 0.724 in differentiating stroke patients from controls. The congruence between their utilization of ophthalmic artery diagnostics and our revised FMD ODM method, underscores the ophthalmic artery's role and potential as a non-invasive access point to the systemic vascular system. This offers a promising pathway for the early detection of conditions such as carotid artery stenosis.”

# Reviewer report: Reviewer response for version 1

**DOI:** 10.5256/f1000research.180947.r408440

**Published:** 2025-09-24

**Authors:** Andrew Tirsi

**Affiliations:** 1 Donald and Barbara Zucker School of Medicine at Hofstra/Northwell, Hempstead, New York, USA

This is a very interesting report describing the use of a new Falck FMD and its use in screening "normal" patients with no overt signs of vascular abnormalities but at high risk  for future ischemic events.

 This patient was considered healthy, until MCRAP and OPP were measured using this non-invasive device.

 What is missing is obstructive sleep apnea status which could also show some changes in OPP but in this case, these changes could manifest differently and most likely binocularly.

 The effects of amlodipine on OPP could also be discussed here.

 I would like to recommend adding a figure showing the screen of the device with all the measurements, and maybe a short explanation of how the measurement is done, since no literature is available about this device.

Are enough details provided of any physical examination and diagnostic tests, treatment given and outcomes?

Yes

Is the case presented with sufficient detail to be useful for other practitioners?

Yes

Is sufficient discussion included of the importance of the findings and their relevance to future understanding of disease processes, diagnosis or treatment?

Yes

Is the background of the case’s history and progression described in sufficient detail?

Yes

Reviewer Expertise:

Normal tension glaucoma, OPP, electrophysiology in glaucoma, retinal ganglion cell dysfunction in glaucoma, mitochondrial dysfunction in glaucoma and other retinal diseases

I confirm that I have read this submission and believe that I have an appropriate level of expertise to confirm that it is of an acceptable scientific standard.

# Response

**Published:** 2025-10-06

**Authors:** Anny Cheng

**Affiliations:** Ophthalmology, University of Florida, Gainesville, Florida, USA

We thank you and the reviewers for your valuable comments. We have revised the manuscript accordingly and provided the following point-to-point responses:

**Reviewer 1 Comments:**

**Comment #1:** What is missing is obstructive sleep apnea status which could also show some changes in OPP but in this case, these changes could manifest differently and most likely binocularly.

 The effects of amlodipine on OPP could also be discussed here.

**Reply:** We discussed the effect of Obstructive sleep apnea and amlodipine on OPP in Discussion Section, 2 ^nd^ paragraph “Notably, systemic blood pressure may significantly influence OPP.​  A study reported that a nocturnal dip in blood pressure, characterized by a systolic reduction of 10-12% and a diastolic reduction of 14-17%, has been linked to compromised OPP. ^6^ Patients receiving aggressive antihypertensive medication that leads to excessive blood pressure lowering are also at risk of low OPP. If our patient's systemic blood pressure was not appropriately controlled with amlodipine, it could paradoxically lead to an increase in OPP, potentially masking cardiovascular disease diagnoses. The relationship between OPP and ocular blood flow is complex, largely due to the eye's autoregulation mechanisms. However, vascular endothelial dysfunction, such as that seen in obstructive sleep apnea (OSA), can impair these autoregulatory capabilities, leading to unstable OPP and significantly impacting ocular perfusion. ^7^ “

*6. Schmidl D, Garhofer G, Schmetterer L. The complex interaction between ocular perfusion pressure and ocular blood flow - relevance for glaucoma. Exp Eye Res. 2011;93(2):141-155. doi:10.1016/j.exer.2010.09.002*

*7.Faridi O, Park SC, Liebmann JM, Ritch R. Glaucoma and obstructive sleep apnoea syndrome. Clin Exp Ophthalmol. 2012;40(4):408-419. doi:10.1111/j.1442-9071.2012.02768.x*

**Comment #2:**I would like to recommend adding a figure showing the screen of the device with all the measurements, and maybe a short explanation of how the measurement is done, since no literature is available about this device.

**Reply:** we added a figure and explanation showing the screen of the device with all the measurements per request.
